# The role of neutrophils in the pathophysiology of inflammatory bowel diseases

**DOI:** 10.1111/prd.70037

**Published:** 2026-04-09

**Authors:** Joao Paulo Steffens, Ekin Yay‐Kus, Rafael Scaf de Molon, Viorelia Stoica, Peter Rimmer, Josefine Hirschfeld, Asif Jilani Iqbal, Tariq Iqbal, Iain Chapple

**Affiliations:** ^1^ School of Health Sciences, College of Medicine and Health University of Birmingham Birmingham UK; ^2^ Department of Stomatology Federal University of Paraná Curitiba Paraná Brazil; ^3^ Birmingham NIHR Biomedical Research Centre in Inflammation Birmingham UK; ^4^ Department of Diagnosis and Surgery Araçatuba School of Dentistry, Sao Paulo State University—UNESP Araçatuba São Paulo Brazil; ^5^ Department of Microbiology and Infection University of Birmingham Birmingham UK; ^6^ Department of Cardiovascular Sciences University of Birmingham Birmingham UK

**Keywords:** cell biology, clinical laboratory techniques, endocrinology, inflammatory bowel diseases, medical laboratory science, neutrophils

## Abstract

**Objectives:**

Inflammatory bowel disease (IBD) encompasses a spectrum of chronic disorders of the gastrointestinal tract, with a potential bidirectional relationship with periodontitis. Neutrophils are key regulators of immune‐inflammatory responses and play a major role in both diseases. Isolating and characterizing gut lumen neutrophils may help to map the evolution of cell phenotypes from peripheral blood to saliva and help explain certain mechanistic relationships within the oral‐gut axis. This review aims to critically evaluate the biological sources of human neutrophils and the emerging analytical approaches to their study in IBD.

**Materials and Methods:**

Studies employing various methodological strategies to isolate and analyze neutrophils derived from both systemic (peripheral blood) and mucosal compartments in IBD are synthesized. Data obtained through different analytical modalities are discussed.

**Results:**

Neutrophils play multifaceted roles in IBD beyond their traditional function in pathogen clearance and acute inflammation. They contribute to both tissue injury and repair through the release of proteolytic enzymes, reactive oxygen species, cytokines, and neutrophil extracellular traps. Recent advances in analytical technologies have uncovered remarkable phenotypic and functional diversity, shaped by the local microenvironment within the intestinal mucosa.

**Conclusions:**

Neutrophils' ability to both exacerbate mucosal damage and facilitate resolution of inflammation underscores the need for improved methodological approaches that enable precise characterization of their functional states in both systemic and tissue contexts.

**Clinical Relevance:**

Improved phenotypic and functional profiling of neutrophils may facilitate the identification of biomarkers predictive of disease activity, treatment response, and relapse risk, and contribute to the understanding of the role of neutrophils in the interplay between IBD and periodontitis.

## 1 INTRODUCTION

Inflammatory bowel disease (IBD) encompasses a group of chronic, relapsing inflammatory disorders of the gastrointestinal (GI) tract, principally represented by Crohn's disease (CD) and ulcerative colitis (UC). These conditions are characterized by dysregulated immune and inflammatory responses to the intestinal microbiota, leading to tissue damage, compromised barrier integrity, and altered mucosal homeostasis. While the precise etiology of IBDs remains incompletely understood, it is well established that the interplay between the innate and adaptive immune system plays a fundamental role in IBD pathogenesis. Among the key cellular components of the innate immune system, neutrophils are increasingly recognized not only as effectors of acute inflammation but also as modulators of chronic mucosal injury in IBD.^1^, ^2^, ^3^

Extraintestinal manifestations of IBD may be evident in up to 50% of patients and include other GI (primary sclerosing cholangitis [PSC]), mucocutaneous (erythema nodosum, pyoderma gangrenosum, oral aphthous ulcers, Sweet's syndrome, and orofacial granulomatosis), musculoskeletal (peripheral and axial arthritis and enthesitis), ocular (episcelritis, scleritis, and anterior uveitis), pulmonary (pneumonitis) and vascular (cardiovascular disease, thromboembolism and portal vein thrombosis) organ systems.^4^ In addition, IBD has been independently linked to several other inflammatory diseases, including dental plaque biofilm‐related diseases such as periodontitis.^5^, ^6^

Periodontitis is a ubiquitous chronic inflammatory noncommunicable disease (NCD) that affects ~45%–50% of adults worldwide,^7^ and together with dental caries, accounts for more years lived with disability than any other human disease.^8^ It leads to progressive destruction of the tooth‐supporting structures, ultimately causing tooth loss, impaired speech, compromised nutrition, and reduced quality of life. Its prevalence reflects marked social and economic inequalities, contributing to a substantial public health and financial burden.^9^ The pathogenesis of periodontitis is complex and multifactorial, arising from a dysbiotic shift in the subgingival microbiota that triggers an exaggerated and non‐resolving host immune‐inflammatory response. Bacterial species, such as *Porphyromonas gingivalis*, *Tannerella forsythia*, and *Treponema denticola* interact with host immune cells, leading to the release of pro‐inflammatory cytokines, chemokines, and matrix‐degrading enzymes that mediate connective tissue breakdown and alveolar bone resorption. Genetic, environmental, and behavioral factors, such as smoking, diabetes, and stress (without coping behaviors), further modulate disease susceptibility and progression.^10^, ^11^, ^12^ Beyond its local effects, periodontitis is independently associated with multiple other NCDs, including cardiovascular disease, diabetes mellitus, chronic kidney disease, and adverse pregnancy outcomes, and has been linked to increased all‐cause and premature mortality.^13^ The periodontal bacteremia that arises during routine oral functions, such as chewing, speaking, and toothbrushing can induce systemic inflammation and vascular endothelial dysfunction, contributing to the significant impact of periodontitis upon overall health and well‐being.^14^, ^15^, ^16^, ^17^

**FIGURE 1:**
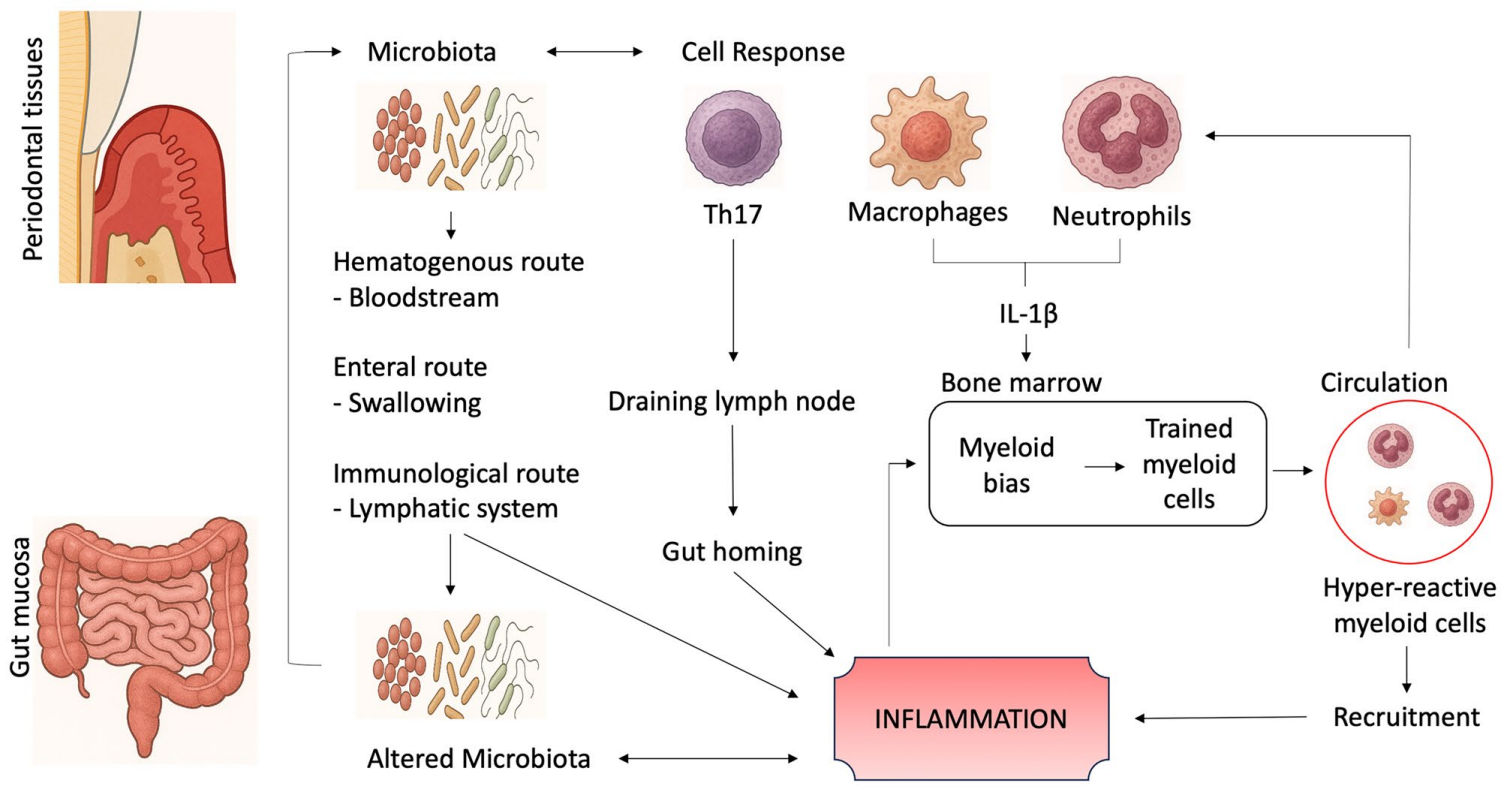
Schematic representation of the potential interaction mechanisms between the periodontal tissues and the gut mucosa. Periodontal microbiota may lead to altered gut microbiota through hematogenous, enteral, or immunological routes. Associated cell responses include gut homing of Th17 cells and trained immunity, where hyperreactive myeloid cells are stimulated. In turn, an altered microbiota and inflamed gut mucosa leads to a feedback loop of perpetuated microbial dysbiosis and myeloid bias. Specifically, neutrophils and other myeloid cells such as macrophages release cytokines such as IL‐1β in response to pro‐inflammatory signals from the systemic circulation. Epigenetic and metabolic alterations in hemopoietic stem cells are induced, leading to enhanced production and release of hyperreactive myeloid cells that promote inflammation when challenged. In the case of neutrophils, hyperreactivity to altered microbiota may include cytokine release,^16^ neutrophil extracellular trap (NET) formation, reactive oxygen species (ROS) release and degranulation, all of which are known to cause collateral host tissue damage.

Potential mechanisms underlying the possible bidirectional relationship between IBD and periodontitis include trained innate immunity during myelopoiesis within the bone marrow and other pathophysiological relationships now embraced by the concept of a direct oral‐gut axis. As discussed later, these include swallowed salivary oral pathogens and also their dissemination via the bloodstream or lymphatic systems to peripheral organs.^18^ Here, neutrophils act as key regulators of the inflammatory pathway and play a major role in the pathogenesis of both diseases due to alterations in their number, activity, interactions with the adaptive immune system, and their reactivity to microbial challenge.^19^, ^20^, ^21^ Briefly, the altered periodontal microbiome within the periodontium elicits diverse cellular responses that include Th17 cells, macrophages, and neutrophils. Th17 lymphocytes drain via lymph nodes to reach the gut mucosa through a process called “gut homing,” which contributes to GI inflammation. Macrophages and neutrophils, in turn, create a pro‐inflammatory environment that leads to a myeloid bias in the bone marrow, and consequently, increased hyperreactive granulocytes within the circulation, contributing to gut mucosal inflammation in a cyclical manner.^17^, ^18^, ^19^, ^20^ A schematic representation of potential interaction mechanisms between the mouth and the intestines is shown in Figure 1.

Neutrophils are first responders, protecting against pathogens by acting as phagocytes; modulating adaptive B‐cell immunity; and altering their survival and functional versatility under certain conditions.^22^ They can also be primed and activated in the circulation to release excess reactive oxygen species (ROS) and cytokines into the extracellular milieu in response to periodontal bacteria, inducing collateral tissue damage upon tissue entry.^16^, ^22^, ^23^ In intestinal inflammation, neutrophils are recruited to the gut via chemokines released by cells such as macrophages, Th‐17 cells, bacterial products, and chemotaxins released by the intestinal epithelium.^24^ Upon sensing infection or tissue injury, they are rapidly mobilized from the bone marrow into the circulation and subsequently recruited to affected sites. There, they deliver a multifaceted antimicrobial repertoire, including phagocytosis, degranulation, production of ROS and cytokines, and release of neutrophil extracellular traps (NETs), all of which constitute the first line of defense against pathogens.^25^, ^26^, ^27^, ^28^ These mechanisms are tightly regulated and highly efficient, allowing neutrophils to swiftly neutralize a broad array of challenges to the integrity of the body's internal systems.

Within IBD, UC, and CD may present different neutrophil phenotypes: while UC is characterized by neutrophil overactivation, including higher neutrophil to total leukocyte ratios and increased release of NETs, ROS, and proteases, CD is characterized by neutrophils with impaired function (lower ROS production and decreased phagocytic activity).^29^ A comparable hyperreactive neutrophil phenotype has also been consistently described in periodontitis.^22^ Patients with periodontitis exhibit peripheral blood neutrophils (PBNs) with exaggerated effector responses, including enhanced NET formation, increased ROS and cytokine production, and heightened degranulation following microbial or cytokine stimulation.^15^, ^22^ This hyper‐NETotic and hyperreactive state mirrors the neutrophil overactivation observed in UC, suggesting that excessive neutrophil activation may represent a shared pathological signature across intestinal and oral mucosal inflammatory diseases.^1^, ^28^ Importantly, periodontal inflammation has been shown to systemically prime circulating neutrophils, rendering them more reactive upon secondary challenge at distal tissue and organ sites,^15^, ^20^ thereby providing a plausible mechanistic link between oral inflammation, altered neutrophil phenotypes, and systemic inflammatory conditions such as IBD.

Interestingly, neutrophils from the gut lumen can reversely transmigrate into the bloodstream, which enhances their lifespan and capacity to produce superoxide radicals.^29^ In periodontitis, chronic inflammation is similarly characterized by a sustained and high‐volume trafficking of neutrophils from the circulation into the periodontal tissues and gingival sulcus, driven by persistent microbial and inflammatory stimuli.^18^, ^19^ Prolonged engagement with the periodontal inflammatory microenvironment has been shown to systemically prime these neutrophils, contributing to the enrichment of hyperreactive populations within the peripheral blood compartment.^15^, ^20^, ^22^ These observations are consistent with altered dynamics of neutrophil trafficking and enhanced functional potential following mucosal tissue engagement, aligning with the reverse transmigration described in gut lumen.

Neutrophils have traditionally been regarded as quintessential effector cells of the innate immune system. Recent technological advances, particularly in single‐cell transcriptomics and multiparameter flow cytometry, have revealed that neutrophils are not a homogenous population of short‐lived, terminally differentiated cells as previously believed. Instead, they represent a phenotypically and functionally diverse population that exhibits remarkable plasticity and context‐dependent adaptation.^30^ Distinct neutrophil subpopulations have been identified based on their maturity, activation state, expression of interferon (IFN)‐stimulated genes (ISGs), and senescence‐associated markers.^31^ Under pathological conditions, including infection, chronic inflammation, autoimmunity, and malignancy, additional specialized subsets emerge. Therefore, neutrophils may exhibit varying levels of differentiation and phenotypic characteristics in blood and at the inflammatory site.^32^

**FIGURE 2:**
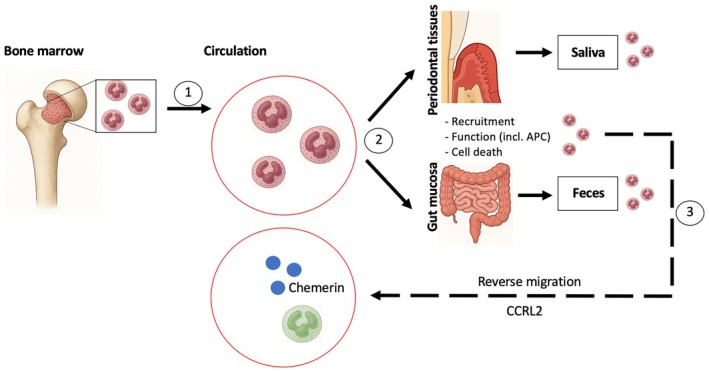
The “natural history” of neutrophils. (1) following their formation in the bone marrow neutrophils are released in circulation; (2) neutrophils are recruited to specific sites, such as periodontal tissues or gut mucosa, where they may exert their functional repertoire (including acting as antigen‐presenting cells), undergo cell death, or be “washed out” with local fluids (e.g., saliva or feces); (3) an alternate fate to neutrophils (dashed lines) is to reversely migrate back into the circulation, in a process mediated by the interaction of chemerin with C‐C motif chemokine receptor‐like 2 (CCRL2).

The majority of phenotyping and characterization of neutrophils in IBD comes from preclinical evidence and peripheral blood in humans, which may not represent their status in situ within peripheral tissues and organs. The natural history of neutrophils includes formation in the bone marrow and release into the circulation; recruitment to specific sites, such as periodontal tissues or gut mucosa, where they may exert their function (including acting as antigen‐presenting cells [APCs]), undergo programmed and nonprogrammed forms of cell death, or be “washed out” with local fluids (e.g., saliva or feces). An alternate fate to neutrophils is to reversely transmigrate back into the circulation, in a process mediated by the interaction of chemerin with C‐C motif chemokine receptor‐like 2 (CCRL2)^32^ (Figure 2). Importantly, investigations of human gut lumen‐derived neutrophils have been restricted to cell counts and related products, mainly in whole gut lavage fluids.^33^, ^34^, ^35^, ^36^ Isolating and characterizing gut lumen neutrophils and analyzing their activity and reactivity using functional assays, may help map the comprehensive evolution of cell phenotype from the peripheral blood to saliva and the intestines in individuals with or without IBD, and contribute to the understanding of the oral‐gut axis associations.

In this review, we explore the potential biological sources for neutrophil characterization in IBD patients and highlight the urgent need for standardized protocols to advance neutrophil phenotyping. Given the shared immunoinflammatory features and microbial dysbiosis observed in IBD and periodontitis, a deeper understanding of neutrophil dynamics could bridge mucosal immunology across gut and oral tissues, paving the way for integrated therapeutic strategies.

## 2 INFLAMMATORY BOWEL DISEASES

### 2.1 Definition

IBD is an autoimmune disease characterized by chronic intestinal inflammation. This condition is an overarching term that encompasses two main groups.^37^ One form is CD, which can affect the entire GI tract from the anus to the oral cavity. Histologically, it is characterized by a patchy pattern of transmural inflammation with granulomas. Another form is UC, which mainly affects the large bowel as a continuous type of inflammation starting from the rectum and extending proximally. Histologically, it is limited to the mucosa and is characterized by crypt abscesses. Extraintestinal manifestations are common.^38^, ^39^

IBD is characterized by a relapsing–remitting pattern with remission phases followed by flare‐ups.^40^, ^41^ The repetitive episodes of inflammation are intrinsically connected to the gut‐oral axis and have a multifactorial etiology. This includes the microbiome composition and the lack of microbiome diversity, host–microbiome interactions, and an abnormal host immune response, together with environmental and genetic factors.^42^, ^43^ Environmental factors like smoking, infections, specific processed foods, and the use of antibiotics and nonsteroidal anti‐inflammatory drugs contribute to the onset of the disease.^44^

### 2.2 Epidemiology

The incidence and prevalence of IBD is on the rise.^45^ Initially considered a common disease in North America and Europe, IBD cases in adults are increasing worldwide, especially in the developing world.^46^ The burden of disease is also increasing in children, adolescents, and the elderly.^47^ Overall, the number of IBD cases has increased from 3.32 million in 1990 to 4.9 million in 2019^46^ with a further projected rise by 2030.^48^ The incidence rates vary geographically. In Europe and North America, the estimates range from 7.3 to 46.12 per 100 000, while the lowest rates are reported in Asia and the Middle East. The prevalence rates range from 187 to 832 in Europe and North America, while the lowest values are observed in South America.^46^ At the same time, increasing global burdens of other NCDs have been observed, including periodontitis.^49^ Many of these are thought to share common risk factors and disease mechanisms. It is therefore important to conduct carefully designed studies to better understand possible disease interactions and associations.

Risk factors include age, with rising rates of pediatric onset IBD and a rising incidence in an older group of adults, over 60s.^50^, ^51^ Smoking is a controllable risk factor for CD, as previous studies showed that cigarette smokers had an increased risk of developing CD, but a reduced risk of developing UC.^46^, ^52^ Family history is also important, with family clustering noted with propensity to develop IBD in susceptible patients that have a positive family history of disease.^53^ Ethnicity‐associated variance in disease phenotypes and demographics is observed even among populations that are geographically co‐located.^54^ There is no consistent significant sex difference.

### 2.3 Clinical features

Symptoms of IBD include abdominal pain, urgency, diarrhea (defined by loose to watery stools), mucous or blood in stool, fatigue, and weight loss.^37^ It is also associated with extraintestinal manifestations, like ankylosing spondylitis, Sjogren Syndrome, and PSC.^4^ In fact, PSC‐IBD is currently a focus of ongoing research to possibly redefine it as a distinct form of disease.^55^

CD is more frequently associated with systemic symptoms including weight loss, oral ulceration, and fatigue.^56^ In those with more aggressive disease phenotypes, complications of penetrating disease, such as abdominal fistulae (e.g., entero‐enteric), abscesses, and bowel perforation may occur. Patients may also separately manifest perianal fistulation and abscesses. Upper GI CD can present symptoms including nausea, emesis, and epigastric pain.^57^ By comparison, patients with UC present more prominent lower GI symptoms with fecal urgency, nocturnal bowel opening and per rectal blood is more common.^56^

Also, mucocutaneous lesions are related to IBD. Erythema nodosum is usually associated with CD and pyoderma gangrenosum coincides mainly with UC.^58^ A series of other autoimmune diseases have been observed to develop concurrently with IBD, like Type 1 diabetes, psoriasis, multiple sclerosis, autoimmune thyroiditis, gluten‐related disorders, and periodontitis, suggesting that there are genetic factors or common risk factors that increase individual susceptibility to those conditions.^59^, ^60^, ^61^

### 2.4 Clinical investigations

The diagnostic pathway involves blood tests, stool samples for fecal calprotectin (FC) and stool polymerase chain reaction (PCR) to exclude an infectious cause of disease, including various infectious gastroenteritides.^56^ The stool test for FC is a valuable marker of inflammation for the diagnosis of IBD and to distinguish between IBD and non‐IBD conditions. It is also used for monitoring disease activity, recurrence, and postoperative relapse.^54^ Further investigations include ileocolonoscopy and imaging.

FC is a protein primarily found in the cytosol of neutrophils and macrophages.^62^ Studies show a high, though variable sensitivity of FC for IBD and a somewhat lower specificity with endoscopic scores of mucosal inflammation.^63^, ^64^ Other GI conditions like colorectal cancer, diverticulosis, and microscopic colitis can also trigger an elevated FC.^65^ Two consequential elevated FC tests increase the diagnostic and monitoring accuracy of IBD.^66^ The cut‐off differs in various studies, ranging from 50 to 200 μg/g.^67^, ^68^ Thus, FC is a relevant, noninvasive tool for diagnosis, monitoring disease activity, and treatment response.^69^

Further clinical investigations include a full blood count to exclude anemia and systemic markers of inflammation such as C‐reactive protein (CRP) and erythrocyte sedimentation rate (ESR). After the initial blood and stool testing, endoscopy and imaging tests follow.^70^ The disease activity is characterized by specific clinical scores and objective indicators such as biomarkers and endoscopic scores. Instruments to assess CD activity are clinical activity indices like the Crohn's disease activity Index (CDAI), the Harvey Bradshaw Index (HBI), and the short CDAI, which is a simplified version. Apart from the clinical evaluation, endoscopic scoring systems to assess levels of inflammation include the Simple Endoscopy Score for Crohn's Disease (SES‐CD), which focuses on mucosal appearance, and the Rutgeerts' score, which assesses the postoperative recurrence of CD.

In the case of UC, the Mayo score is an instrument used to measure disease activity in clinical trials.^71^ For UC, the ulcerative colitis endoscopic index of severity (UCEIS) is used to assess the degree of inflammation (vascular pattern, bleeding, erosions/ulcers) during endoscopy.^72^, ^73^ Apart from these indices, magnetic resonance imaging and magnetic resonance enterography with bowel wall contrast enhancement allow evaluation of wall thickening, edema, and strictures of the small bowel.^37^ Depending on the local capabilities, abdominal ultrasound is increasingly a cost‐effective and significant tool to identify active inflammation in IBD,^74^ while video capsule endoscopy has been proposed for both diagnosis and risk stratification.^75^ Once a diagnosis has been established, the Montreal classification can be used to categorize patients based on age, local manifestations, and disease progression.^76^, ^77^, ^78^

### 2.5 Biological mechanisms

The pathophysiology of IBD is a complex process. An interaction between genes, the immune system, and environmental triggers, including the microbiome and the host response, leads to the onset of disease.^79^ The intestinal mucosa has both a nutrient absorption function and acts as a barrier between the lumen and the lamina propria. It consists of epithelial cells connected by intercellular desmosomal junctions, which interact with the intestinal microbiome and the host immune system. The defects in intestinal epithelial barrier function, either as a primary or secondary loss of function, result in continued deterioration of the mucosa and exposure to the intestinal microbiome. This, in turn, leads to deficient protective mechanisms, such as mucus production and secretion of defensines with intrinsic antimicrobial activity.^80^ To date, it is unclear if mucosal damage precedes or succeeds the inflammatory process. Purportedly, the disbalance ensues as a disruption of tolerance toward commensal microorganisms or an impaired immune response.^80^, ^81^

Beneath the epithelium, the lamina propria contains stromal cells, including fibroblasts, myofibroblasts, and perivascular pericytes, which participate in fibrosis and wound healing. Throughout the inflammatory process, these functions are impaired. Histopathological assessment reveals infiltration of the lamina propria with neutrophils, macrophages, dendritic cells (DC), and natural killer (NK) cells. This, in turn, leads to increased levels of pro‐inflammatory mediators such as tumor necrosis factor (TNF)‐α, Interleukin (IL)‐1β, IFN‐γ, among other cytokines.^80^, ^82^ The immune system comprises adaptive and innate immune arms. The latter, which includes immune innate cells, such as neutrophils, monocytes, macrophages, and DCs, triggers a rapid and nonspecific immune response and facilitates phagocytosis by generating cytokines and chemokines. They can also stimulate the adaptive immune response by presenting antigens,^82^, ^83^ in association with their Major Histocompatibility Complex (MHC)‐II.

### 2.6 Current therapeutic approaches

The ultimate therapeutic goal in IBD is to establish deep remission, with normalization of inflammatory markers, endoscopic and histological healing, resolution of clinical symptoms, and normalization of quality of life.^84^ In the clinical environment where resource limitations exist, these lofty goals are sought in a stepwise fashion and often rely upon surrogate markers.^85^ While conventional treatments include aminosalicylates, corticosteroids, immunomodulators, and advanced therapies, the evolution of treatment strategies and therapeutic paradigms mandates that the treatment of CD and UC be considered separately.

Among patients with UC, 5‐aminosalicyclates—such as mesalazine—remain the first line of therapy. They are useful in the induction or maintenance of remission in mild‐to‐moderate inflammation. The exact working mechanism is unknown, but they seem to modulate the inflammatory response by potentially interfering with the arachidonic acid metabolic pathway and decreasing the synthesis of prostaglandins and leukotrienes. Other mechanisms include the inhibition of TNF‐α, reducing nuclear factor kappa B (NF‐kB) and influencing cellular functions of immune cells (lymphocytes, macrophages, NK cells).^86^ For those with more severe or persistent inflammation, short‐term step‐up escalation therapy includes either systemic or topical corticosteroids.^87^, ^88^ These are utilized to induce symptomatic remission^89^ as a bridge to steroid‐sparing approaches, including advanced therapies that can sustain long‐term remission. The mode of action of corticosteroids involves binding to receptors of the pro‐inflammatory transcription factors and inducing apoptosis of inflammatory cells and activated lymphocytes.^90^, ^91^, ^92^ Through gene expression regulation, cytokines IL‐1β, TNF‐α, IL‐6, IL‐8, and granulocyte‐macrophage colony‐stimulating‐factor (GM‐CSF) are reduced and the synthesis of immunomodulatory cytokines IL‐2, IL‐3, IL‐4, IL‐5, IL‐10, IL‐12, and INF‐γ is inhibited. This leads to a wide blockade of the immune system^90^, ^91^ and an increased risk of opportunistic systemic infections. Systemic corticosteroids have oral and intravenous forms, whereas topical steroids have two applications—one for ileal and one for colonic disease.^89^ In UC, surgical approaches have traditionally been reserved for those with disease refractory to medical therapies, though there is a growing interest in the utility of therapeutic appendicectomy as a surgical strategy that can be employed at an earlier stage.^93^, ^94^

In patients with moderate‐to‐severe CD, the importance of early aggressive medical therapy with a top‐down approach is well established. This may involve the initial use of corticosteroids, but only as a bridge to the urgent initiation of advanced therapies, ideally before the establishment of fibrotic or penetrating symptoms.^95^, ^96^ Furthermore, surgical approaches may be employed as a complementary or as a first‐line therapy in selected cases.^97^

Across UC and CD, therapeutic options for induction and maintenance of remission include immunomodulators and advanced therapies.^98^ An increasing arsenal of advanced therapies is available including TNF inhibitors (adalimumab, infliximab, certolizumab, golimumab), integrin antagonists (vedolizumab), IL antagonists (IL12/23—ustekinumab; IL23—risankizumab, guselkumab, mirikizumab), JAK inhibitors (tofacitinib, filgotinib, upadacitinib) and S1P receptor inhibitors (ozanimod, etrasimod). While a more prominent role remains as a potential monotherapy in UC, the use of immunomodulators such as azathioprine in CD is primarily with a view to the prevention and suppression of anti‐drug antibodies to anti‐TNF medications.^99^, ^100^ The benefit of the combination of anti‐TNF medications and Azathioprine has been shown across both UC and CD.^101^, ^102^ Therapeutic sequencing, both in terms of first‐line agent and among those with loss of response to advanced therapies, should be individualized, but decisions are supported by guidelines and large real‐world datasets.^103^ With the advent of biosimilar medicines, now therapies such as Infliximab are off‐patent, and financial considerations are also important when making decisions across financially stretched health services. Novel drugs and therapies, like fecal microbiota transplantation, are emerging.^104^, ^105^, ^106^, ^107^, ^108^ At present, evidence for therapeutic efficacy is largely limited to UC, with more conflicting results observed among those with CD.

In vitro and animal studies have also targeted neutrophils for IBD treatment using different classes of drugs, including: (a) specialized pro‐resolving mediators (SPMs), to regress intestinal inflammation; (b) peptidylarginine deiminase 4 (PAD4) inhibitors, to target NETs; (c) microbial metabolites, to suppress neutrophil migration and NET formation; (d) artemisinin analogues, to inhibit immune cells including neutrophils; (e) immunosuppressant (cyclosporine), to regulate neutrophil function; (f) classic drugs (N‐acetylcysteine and 5‐amino salicylic acid) to regulate the release of ROS from neutrophils; and (g) myeloperoxidase (MPO) inhibitors, for their anti‐inflammatory and antioxidant activity.^109^

## 3 NEUTROPHIL BIOLOGY

### 3.1 Neutrophils in bridging innate and acquired immunity

Neutrophils, constituting ~40%–70% of circulating leukocytes in humans, represent one of the most abundant and critical components of the innate immune system. As frontline defenders, they are rapidly mobilized in response to invading pathogens or tissue injury, mounting immediate and potent antimicrobial responses.^110^ They express a diverse repertoire of pattern recognition receptors (PRRs), including toll‐like receptors (TLRs), complement receptors, and Fc receptors. This equips them to detect a wide range of pathogen‐associated molecular patterns (PAMPs) and damage‐associated molecular patterns (DAMPs), enabling them to initiate and amplify inflammatory responses through the release of cytokines and chemokines.^3^, ^111^, ^112^ Once activated, neutrophils undergo a potent respiratory burst, mediated by the NADPH oxidase complex. This process rapidly consumes molecular oxygen to generate superoxide anions and other downstream ROS, which exert direct microbicidal activity. These ROS act synergistically with enzymes stored in azurophilic granules, such as neutrophil elastase and MPO, to degrade engulfed pathogens. Moreover, these granules contribute to the formation of NETs.^1^, ^27^, ^48^, ^111^, ^113^, ^114^, ^115^

For many years, neutrophils were considered short‐lived and terminally differentiated leukocytes with a limited role confined to the early stages of inflammation. However, accumulating evidence over the past decade has fundamentally transformed this view. It is now clear that neutrophils play critical roles beyond the innate immune frontlines, actively contributing to the regulation and shaping of adaptive immunity. One of the key mechanisms through which neutrophils influence adaptive responses is by secreting a complex repertoire of cytokines, chemokines, and alarmins. These molecules modulate the activation, maturation, and recruitment of various immune cells, including macrophages, DCs, NK cells, and lymphocytes.^27^ As such, neutrophils serve not only as executioners of pathogen clearance but also as orchestrators of broader immune responses, bridging the gap between innate detection and adaptive specialization. Chronic mucosal inflammatory conditions like periodontitis, characterized by persistent microbial challenge and tissue damage, have been linked to context‐dependent diversification of neutrophil phenotypes, reinforcing the concept that neutrophil heterogeneity is influenced by local inflammatory signals.^1^, ^18^, ^19^

A particularly important aspect of neutrophil involvement in adaptive immunity lies in crosstalk with professional APCs, such as DCs and macrophages. Neutrophils release chemokines, including CCL3, CCL4, and CCL5, that attract immature DCs to sites of infection or inflammation, thereby facilitating the uptake of antigens and enhancing the initiation of adaptive immune responses.^27^ Furthermore, neutrophils can release extracellular vesicles (EVs) and NET components that carry antigenic material. These elements can be internalized by DCs and macrophages, leading to enhanced antigen processing and presentation to CD4^+^ and CD8^+^ T cells.^25^, ^112^, ^113^, ^116^ This mechanism has been corroborated in animal models of infection, such as those involving *Leishmania major* and *Mycobacterium tuberculosis*, where the depletion of neutrophils impairs DC migration to draining lymph nodes and diminishes subsequent T‐cell priming.^112^ These findings highlight the indispensable role of neutrophils in shaping the early events of adaptive immunity and indicate that comparable neutrophil‐APC interactions operate within periodontal tissues, where chronically activated neutrophils contribute to the modulation of local adaptive immune responses within the gingival mucosa.

In certain inflammatory contexts, neutrophils themselves can acquire antigen‐presenting capabilities. Exposure to pro‐inflammatory cytokines, such as IFN‐γ, GM‐CSF, and TNF‐α, along with microbial stimuli, can induce the expression of MHC‐II and co‐stimulatory molecules (CD80 and CD86) on the surface of neutrophils. Under these conditions, neutrophils can process and present antigens to CD4^+^ T cells. In vitro studies have shown that human neutrophils are capable of presenting viral antigens such as cytomegalovirus pp65 or influenza hemagglutinin to memory CD4^+^ T cells in an MHC‐II‐dependent manner.^112^, ^116^ While these antigen‐presenting neutrophils are generally less efficient than classical DCs, their capacity to contribute to T‐cell activation is particularly relevant in highly inflamed environments or in the context of vaccination, where their presence may enhance the magnitude and specificity of adaptive immune responses.

In addition to their functions at peripheral sites of infection, activated neutrophils are also capable of trafficking to secondary lymphoid organs. After migrating into inflamed tissues, neutrophils can enter draining lymph nodes via afferent lymphatic vessels or through high endothelial venules (HEVs), a process directed by chemokine receptors such as CCR7.^111^ Within the lymph node microenvironment, neutrophils that have experienced tissue‐derived signals can upregulate adhesion molecules and co‐stimulatory markers. Although many of these cells undergo apoptosis upon entry, their interactions with resident immune cells are consequential. Neutrophils in lymph nodes can contribute to antigen delivery, modulate the activation of DCs, and interact directly with T and B cells.^111^, ^113^, ^115^ Additionally, neutrophil cytoplasts, enucleated cellular remnants generated during NET formation, can persist within lymphoid tissues and stimulate DCs to drive T helper cell polarization toward Th17 or Th2 phenotypes,^114^ further demonstrating the nuanced immunoregulatory capacity of neutrophils.

The interplay between neutrophils and B cells reveals another dimension of their immunological versatility. Splenic neutrophils have been shown to support T‐cell‐independent antibody production by interacting directly with marginal zone B cells. Through this interaction, neutrophils can promote class switching and the generation of specific antibodies, highlighting their potential to contribute to humoral immunity. On the T‐cell side, neutrophils influence the differentiation of helper T‐cell subsets through the secretion of cytokines, such as IL‐6, IL‐1β, and IL‐23, all of which promote Th17 lineage commitment. Conversely, neutrophil‐derived IL‐12 supports the differentiation of Th1 cells, illustrating their bidirectional role in shaping T helper cell polarization. Importantly, neutrophils are also capable of restraining excessive immune responses. By expressing immunosuppressive molecules such as programmed death‐ligand 1 (PD‐L1), arginase 1 (Arg1), and through the generation of ROS, neutrophils can suppress T‐cell proliferation and effector functions, thereby maintaining immune homeostasis and preventing overactivation.^25^

Further complexity arises from the recognition of neutrophil heterogeneity. Under specific physiological or pathological conditions, distinct neutrophil subsets with regulatory or suppressive functions can emerge.^30^ These subsets are phenotypically and functionally specialized and may arise in response to local microenvironmental signals. Regulatory neutrophils have been identified in both homeostatic and inflamed tissues, including within secondary lymphoid organs. Periodontitis also provides a clinically relevant example of such environment‐driven neutrophil diversification, where prolonged exposure to microbial dysbiosis and inflammatory mediators is associated with functionally distinct peripheral and tissue neutrophil subsets.^1^, ^15^, ^22^ Their ability to directly engage with adaptive immune cells and modulate immune responses suggests that neutrophil function is highly context‐dependent and cannot be fully appreciated through a unidimensional lens.^117^, ^118^

In summary, the evolving understanding of neutrophil biology has redefined these cells as more than just rapid responders in innate immunity. Instead, neutrophils emerge as dynamic players in the coordination of immune responses across multiple layers. By facilitating the recruitment and activation of APCs, presenting antigens, trafficking to lymphoid organs, enhancing humoral responses, guiding T‐cell differentiation, and exerting immunoregulatory control, neutrophils are central integrators of innate and adaptive immunity. These findings position neutrophils not only as first‐line defenders but also as critical architects of immune system orchestration. Moreover, the recognition of neutrophils as key players in bridging innate and acquired immunity has profound implications for vaccine design, infection control, and the treatment of autoimmune disease, inflammatory disorders, and cancer. By harnessing or modulating neutrophil functions, novel therapeutic strategies may be developed to enhance protective immunity or restore immune tolerance. As the field continues to unravel neutrophil diversity, plasticity, and molecular mechanisms of interaction with adaptive immune cells, the longstanding dogma of neutrophils as short‐lived, homogeneous effectors is giving way to a nuanced appreciation of their multifaceted immunological roles.

### 3.2 Neutrophils and specialized pro‐resolving lipid mediators

Beyond their established roles in driving acute inflammation and bridging innate and adaptive immunity, neutrophils are increasingly recognized as critical regulators of the resolution phase of inflammation. Resolution is not a passive process but an active, highly coordinated program aimed at restoring tissue homeostasis and preventing chronic pathology. Neutrophils contribute to this process through the production of SPMs, a family of bioactive lipid mediators derived from polyunsaturated fatty acids, including lipoxins, resolvins, protectins, and maresins.^119^ These mediators counterbalance the pro‐inflammatory effects of eicosanoids, such as leukotrienes and prostaglandins, orchestrating the clearance of apoptotic cells and promoting tissue regeneration. Importantly, neutrophil‐derived SPMs not only limit excessive tissue damage but also actively engage with macrophages and epithelial cells to facilitate wound repair and mucosal healing.^120^

A central aspect of this resolution program involves the lipid mediator class switch that occurs during the course of inflammation. Early neutrophil infiltration is associated with the generation of pro‐inflammatory prostaglandins, particularly prostaglandin E2 (PGE2), and leukotrienes. As the inflammatory response progresses, PGE2 levels rise and trigger the induction of 15‐lipoxygenase (15‐LOX), a key enzyme that drives the biosynthesis of SPMs such as lipoxin A4 (LXA4).^119^, ^121^ Neutrophils are essential in this transition, as they provide both substrates and enzymatic activity necessary for SPM generation. LXA4, in particular, binds to the N‐formyl peptide receptor 2 (FPR2) expressed on neutrophils and other leukocytes, limiting further neutrophil recruitment and promoting their apoptosis. This feedback loop ensures that neutrophils not only initiate but also actively contribute to the cessation of inflammation.^122^

Therefore, neutrophils serve as key players not only in initiating and amplifying inflammation but also in programming its resolution through the production of pro‐resolving lipid mediators. By generating SPMs in response to elevated PGE2 and other signals, neutrophils coordinate the termination of inflammation, clearance of apoptotic cells, and tissue regeneration.

### 3.3 Characteristics of neutrophils in health and disease

Morphologically, neutrophils are distinguished by their polymorphonuclear, multilobed nuclei and cytoplasm densely packed with granules containing a wide array of antimicrobial proteins and enzymes. These cells arise from hematopoietic progenitors in the bone marrow via a tightly orchestrated process known as granulopoiesis, which is regulated by transcription factors such as CCAAT/enhancer‐binding protein alpha (C/EBPα). Under homeostatic conditions, the human body produces an estimated 5 × 10^10^ to 1 × 10^11^ neutrophils per day to maintain a steady circulating pool. Neutrophils have a relatively short lifespan in circulation, typically ranging from several hours to a few days, a dynamic turnover process confirmed using an in vivo isotope‐labeling technique.^123^

**FIGURE 3:**
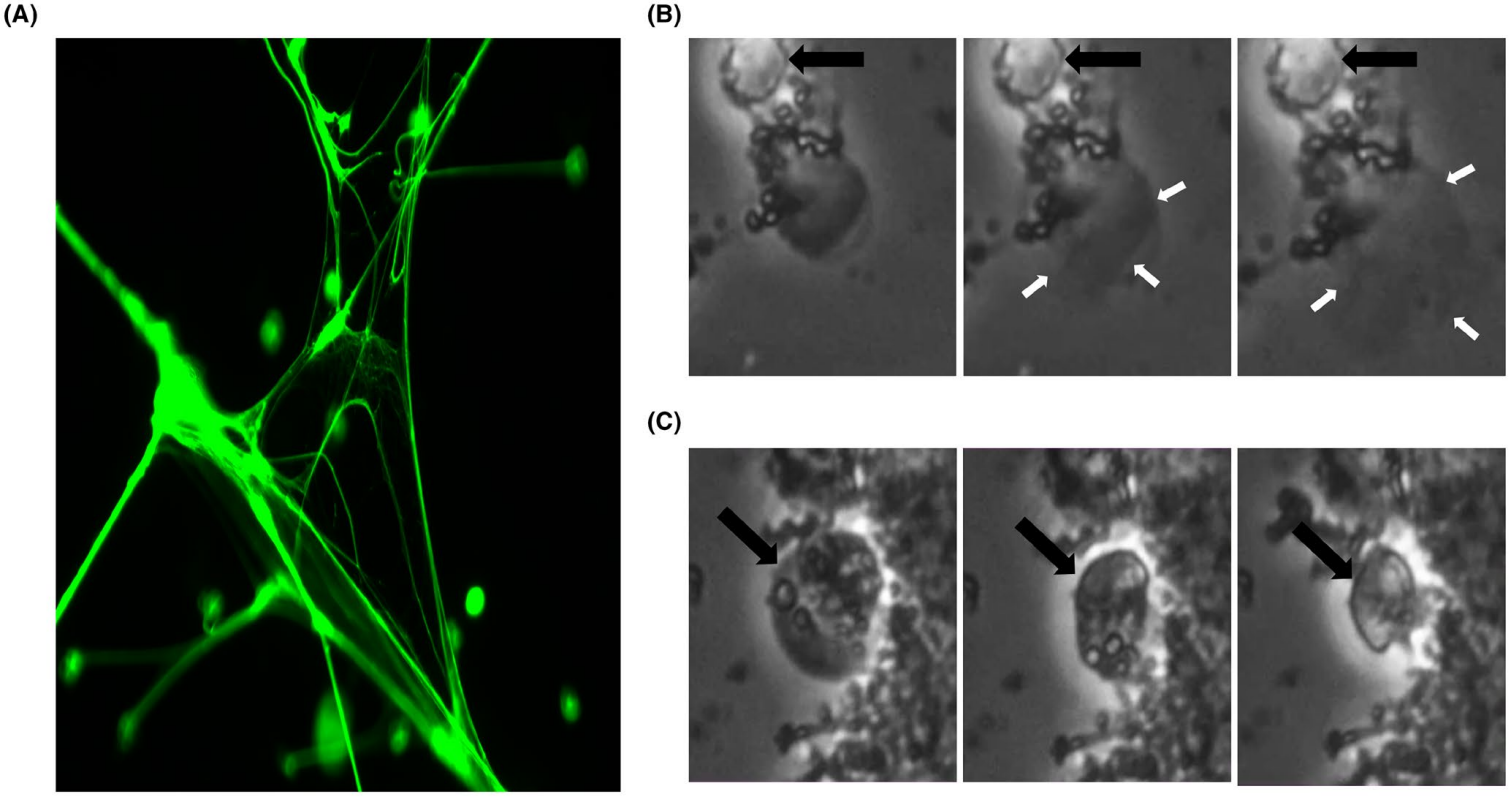
Images of neutrophils (A) releasing neutrophil extracellular traps (NETs) using fluorescent extracellular DNA staining and fluorescent microscopy of neutrophils isolated from healthy human peripheral blood and stimulated ex vivo; (B) undergoing cell death by NETosis, necrosis, or lysis; and (C) performing phagocytosis in dental biofilm surface. Both (B) and (C) are orally migrated neutrophils derived from a small‐volume aspirate of a healthy gingival crevice and imaged immediately for 30 min at 40× magnification. Black arrow: Oral neutrophils. White arrows: Release of cellular contents, possibly including decondensed chromatin and proteins (NETs). Note the “cloudy” area that follows cell death.

Once released into the bloodstream, neutrophils quickly enter a state of immune surveillance. They continuously patrol peripheral tissues, maintaining readiness to respond to microbial invasion or sterile injury. Neutrophils exhibit a canonical functional repertoire that includes chemotaxis, phagocytosis, degranulation, generation of ROS, and the release of NETs. Collectively, these functions enable efficient detection, containment, and elimination of pathogens.^2^, ^123^, ^124^, ^125^, ^126^ Images of neutrophils releasing NETs, undergoing cell death and performing phagocytosis can be found in Figure 3.

Chemotactic responses are driven by the interaction of chemokine ligands with receptors such as CXCR2 and CXCR4, and are further directed by gradients of IL‐8, complement components, leukotrienes, and other inflammatory mediators. Upon arriving at the site of infection, neutrophils engulf microbes into phagosomes that rapidly fuse with azurophilic and specific granules, forming phagolysosomes where potent antimicrobial mechanisms are deployed. Within these compartments, the NADPH oxidase complex initiates a respiratory burst, leading to the production of ROS such as superoxide and hydrogen peroxide. Simultaneously, enzymes such as MPO, neutrophil elastase, cathepsin G, and lactoferrin act synergistically to degrade microbial components and facilitate pathogen clearance.^30^, ^123^, ^127^, ^128^, ^129^

Functionally, neutrophil heterogeneity in different tissues and conditions is reflected in their diverse effector capabilities. In infectious scenarios, some neutrophils exhibit hyperinflammatory profiles, producing pro‐inflammatory cytokines, such as IL‐1, IL‐6, TNF‐α, and chemokines like CCL3 (MIP‐1α), CCL4 (MIP‐1β), and CXCL8 (IL‐8). These mediators enhance pathogen clearance and stimulate other immune cells. Conversely, during systemic or chronic inflammation, neutrophils may shift toward anti‐inflammatory phenotypes, characterized by secretion of IL‐10 and CCL2 (MCP‐1), thereby contributing to immune suppression and resolution of inflammation.^127^ In the tumor microenvironment, neutrophils can either promote tumor progression via angiogenesis, matrix remodeling, and suppression of T‐cell activity, or inhibit tumor growth through cytotoxic mechanisms and antigen cross‐presentation.^124^

Many specific populations of neutrophils have been described in different scenarios. An atypical population of granulocytes with altered buoyancy may co‐exist in certain autoimmune conditions, such as in systemic lupus erythematosus. These low‐density neutrophils (LDNs) are markedly expanded and exhibit heightened capacity to generate NETs. In conjunction with immunostimulatory components, NETs activate plasmacytoid DCs, resulting in elevated type I IFN production and promoting chronic vascular inflammation.^129^ Dysregulation of NETosis—a unique form of cell death through lytic NET formation—has been implicated in tissue injury, autoimmunity, and thrombotic events due to the exposure of immunogenic self‐antigens and pro‐coagulant factors. In physiological settings, aged or senescent neutrophils are characterized by increased surface expression of CXCR4, decreased chemotactic responsiveness, and are ultimately cleared by macrophages in the bone marrow or spleen.^126^ In sepsis, a dysregulated neutrophil response includes the emergence of subsets with impaired microbial killing but elevated immunosuppressive function, contributing to immune paralysis and increased susceptibility to secondary infections.^130^ Similarly, during myocardial infarction, neutrophils infiltrate the infarcted tissue and initially promote inflammation through inflammasome activation and ROS production. As the response evolves, neutrophils can polarize toward a reparative (N2‐like) phenotype, contributing to tissue remodeling and resolution of inflammation.^128^

At the metabolic level, neutrophils display considerable adaptability. While glycolysis is the primary metabolic pathway supporting their energy needs under homeostatic conditions, neutrophils can reprogram their metabolism to utilize oxidative phosphorylation, relying on substrates such as glutamine or fatty acids, particularly in glucose‐limited environments, such as within tumors or chronically inflamed tissues.^126^ This metabolic flexibility is essential for supporting their extended survival, enhancing ROS generation, and modulating their effector functions, including NET formation and cytokine secretion. Dysregulation of these metabolic programs has been linked to pathological conditions, including the hyper‐NETosis seen in diabetes and the emergence of tumor‐promoting neutrophil phenotypes.

From a clinical perspective, the absolute neutrophil count (ANC) serves as a critical biomarker for diagnosing and monitoring various pathological states. Neutrophilia, or elevated neutrophil counts, is typically observed in acute bacterial infections, systemic inflammation, physical stress, trauma, and certain hematological malignancies, such as myeloproliferative disorders. In contrast, neutropenia, a reduction in circulating neutrophils, may result from chemotherapy, bone marrow suppression, or severe infections and is associated with increased susceptibility to opportunistic infections. A so‐called “left shift,” denotes the presence of immature neutrophil forms such as band cells in peripheral blood and is indicative of emergency granulopoiesis and is commonly seen in acute infections.^131^, ^132^

In conclusion, neutrophils are no longer viewed merely as transient preprogrammed foot soldiers of the innate immune response. Instead, they are increasingly recognized as versatile and highly dynamic immune cells with complex roles in both health and disease. In homeostasis, neutrophils are characterized by rapid mobilization, potent antimicrobial activity, efficient clearance mechanisms, and metabolic agility. Under pathological conditions, they diversify into specialized subsets that can either exacerbate or resolve disease processes through context‐dependent effector functions. A deeper understanding of neutrophil heterogeneity, plasticity, and immunoregulatory capacity is essential for developing targeted therapeutic strategies. These may include modulation of neutrophil recruitment, inhibition of pathological NETosis, reprogramming of metabolic pathways, or manipulation of their polarization states to enhance host defense while minimizing tissue damage.

### 3.4 Biological plausibility for a role of neutrophils in IBD

In IBD, neutrophil infiltration of the intestinal lamina propria and epithelial layer is a hallmark feature, especially during active disease. In UC, for instance, the presence of crypt abscesses composed of neutrophils is a diagnostic criterion. Their activation leads to the release of proteolytic enzymes, such as elastase, MPO, ROS, NETs, and pro‐inflammatory cytokines, all of which can contribute to epithelial injury and perpetuation of inflammation.^20^, ^133^

One of the key consequences of excessive neutrophilic infiltration in the gut mucosa is the disruption of the epithelial barrier. Neutrophil‐derived mediators can degrade tight junction proteins and basement membrane components, leading to increased intestinal permeability and exposure of the lamina propria to luminal antigens. This loss of barrier function is not only a consequence but also a driver of chronic intestinal inflammation. In UC, neutrophils are found to actively transmigrate across the epithelium, and this process, while crucial for microbial clearance, also results in epithelial cell death and mucosal erosion.^134^

A comparable pattern of neutrophil‐driven epithelial disruption is observed in periodontitis, where there is a continuous and constitutive flux of neutrophils migrating across the junctional epithelium and into the gingival sulcus as part of immune surveillance processes and subsequent responses to the subgingival biofilm.^18^, ^19^ Unlike the episodic neutrophil transmigration associated with acute intestinal inflammation, this sustained transepithelial migration within periodontal tissues represents a chronic process which, while essential for microbial surveillance, also contributes to epithelial attachment loss, apical migration of the junctional epithelium, and connective tissue breakdown.^18^, ^21^ Thus, in both IBD and periodontitis, excessive or dysregulated neutrophil passage across epithelial barriers constitutes a shared pathogenic mechanism, whereby persistent neutrophil flux compromises barrier integrity and perpetuates mucosal inflammation, despite differences in epithelial structure and function.^1^, ^17^

A growing body of work demonstrates that neutrophils in IBD exist as multiple phenotypically distinct subsets rather than a single uniform population. Classical high‐density neutrophils (HDNs) represent the dominant circulating population and are well characterized in human IBD, where they display heightened activation, increased NET formation, and enhanced FcγR expression.^135^, ^136^ In contrast, LDNs, initially described in systemic autoimmune diseases, have also been identified in active UC and CD, where they exhibit increased spontaneous NETosis and pro‐inflammatory cytokine production.^137^, ^138^ Transcriptional profiling of intestinal biopsies has identified IFN‐stimulated neutrophil states enriched in UC, characterized by ISGs‐high signatures and enhanced antimicrobial and NET‐associated pathways.^139^, ^140^ By comparison, SPM‐producing neutrophil subsets remain largely inferred from murine colitis models and ex vivo lipid mediator studies rather than definitively demonstrated in human IBD tissue.^141^, ^142^ Similarly, hyperinflammatory versus regulatory neutrophil phenotypes, described in cancer and systemic inflammation, have been proposed in IBD based on functional assays showing exaggerated ROS production, impaired efferocytosis, and enhanced NET formation,^135^, ^143^ but stable regulatory neutrophil subsets have not yet been clearly defined in human intestinal mucosa. Together, these observations highlight that while several neutrophil states, particularly activated HDNs, LDNs, and IFN‐high tissue neutrophils, are clearly present in human IBD, other subsets remain extrapolated from related inflammatory diseases or experimental models and require further validation in intestinal biopsies and mucosal aspirates.

#### 3.4.1 Neutrophils and pro‐resolving lipid mediators in IBD

The dysregulation of pro‐resolving pathways has been implicated in the pathogenesis of IBD. Patients with CD and UC exhibit impaired production of lipoxins and resolvins, along with defective signaling through their receptors, leading to a failure in timely resolution of inflammation and perpetuation of mucosal damage.^144^ Experimental models support this notion, as exogenous administration of SPMs accelerates the resolution of colitis, reduces neutrophil accumulation, and enhances mucosal repair.^145^ These findings suggest that the defective resolution machinery in IBD is not merely a bystander effect but a pathogenic mechanism that drives chronicity. Importantly, neutrophil‐derived SPMs not only limit excessive tissue damage but also actively engage with macrophages and epithelial cells to facilitate wound repair and mucosal healing, processes that are of particular relevance in the context of IBD.^120^

In the intestinal mucosa, the actions of SPMs are especially relevant given the persistent antigenic stimulation and microbial load that characterize this environment. Studies have demonstrated that LXA4 and resolvins attenuate neutrophil infiltration into inflamed tissues while enhancing macrophage‐mediated efferocytosis of apoptotic neutrophils, thereby preventing secondary necrosis and the release of tissue‐damaging contents.^146^, ^147^, ^148^ Furthermore, SPMs such as resolvin‐D1 and maresin‐1 have been shown to promote epithelial barrier repair by stimulating epithelial proliferation and tight junction integrity, processes critical for mucosal healing in IBD.^120^, ^149^ Thus, neutrophil‐derived lipid mediators not only restrain inflammation but also provide essential cues for tissue regeneration.

Within periodontal tissues, chronic inflammation is likewise associated with a failure to adequately activate pro‐resolving lipid mediator pathways, resulting in sustained neutrophil recruitment and incomplete resolution of inflammation.^1^, ^17^, ^18^ Reduced local availability of lipoxins and resolvins, together with impaired receptor‐mediated signaling, has been reported in inflamed gingival sites and is accompanied by prolonged neutrophil survival and defective efferocytosis.^116^, ^117^ In experimental models of periodontitis, therapeutic augmentation of pro‐resolution pathways through administration of specialized pro‐resolving lipid mediators dampens neutrophil accumulation, limits connective tissue degradation, and promotes tissue regeneration.^116^, ^117^ Taken together, these observations indicate that disruption of neutrophil‐driven pro‐resolution programs contributes to the chronicity of periodontal inflammation and aligns mechanistically with defective resolution processes described in IBD.^1^, ^17^

In the context of IBD, therapeutic strategies aimed at enhancing neutrophil‐derived resolution pathways hold promise for shifting the inflammatory balance away from chronic damage toward mucosal healing and restoration of intestinal homeostasis.^119^, ^120^

#### 3.4.2 In vitro studies

A substantial body of in vitro research has contributed to elucidating the molecular and cellular mechanisms by which neutrophils participate in the initiation, amplification, and chronic perpetuation of IBD.^150^, ^151^, ^152^ These studies reveal that neutrophils, beyond serving as first responders in mucosal immunity, actively contribute to epithelial barrier disruption and the propagation of inflammatory circuits within the intestinal microenvironment. Furthermore, in vitro studies have provided mechanistic insights into the functional properties of neutrophils in the context of intestinal inflammation. Co‐culture systems involving intestinal epithelial cells (IECs) and neutrophils have shown that activated neutrophils induce epithelial barrier dysfunction, reduce transepithelial electrical resistance, and increase paracellular permeability through the release of ROS and proteases.^153^ Additionally, neutrophils have been shown to enhance epithelial cell apoptosis and proliferation, further contributing to mucosal disruption and defective wound healing.^154^

Neutrophil–epithelial interactions have also been explored utilizing in vitro models to recapitulate the structural and functional properties of the intestinal epithelium and have revealed that neutrophils migrating through epithelial monolayers alter tight junction integrity and stimulate pro‐inflammatory cytokine release, including IL‐8 and TNF‐α.^155^ In vitro, NET‐activated mononuclear cells within the lamina propria produce pro‐inflammatory cytokines via ERK1/2 signaling. Overall, the findings suggest that NETs contribute to mucosal inflammation in UC. The presence of microbial products such as LPS and flagellin can amplify these responses through TLR signaling pathways, mimicking the environment of the inflamed gut in IBD.^155^

Parallel studies employing neutrophils derived from patients with UC have demonstrated an enhanced capacity for NET release upon stimulation with inflammatory mediators such as TNF‐α. These NETs, composed of decondensed chromatin and neutrophil granule proteins, such as MPO, neutrophil elastase, and citrullinated histone H3, exert cytotoxic effects on IECs. They promote epithelial apoptosis, disrupt tight junction assembly, and impair wound healing, thereby exacerbating mucosal injury. Supporting this, proteomic analyses of inflamed colonic mucosa from UC patients consistently demonstrate increased levels of PAD4, MPO, citrullinated histones, and other NET‐associated markers. Interestingly, such upregulation appears more prominent in UC compared with CD, suggesting disease‐specific differences in the nature and extent of neutrophil activation.^83^, ^156^ These NET‐mediated mechanisms of epithelial injury in UC reflect analogous phenomena observed in periodontitis, where excessive neutrophil activation and NET formation contribute to disruption of the gingival epithelial barrier and loss of epithelial attachment.^1^, ^18^ In periodontal tissues, NET‐associated proteases, ROS, and citrullinated histones have been implicated in epithelial cell apoptosis, degradation of junctional complexes, and impairment of wound healing, thereby facilitating connective tissue breakdown and apical migration of the junctional epithelium.^18^, ^21^, ^22^ Thus, although the target tissues differ anatomically, NET‐driven epithelial damage represents a shared pathological mechanism linking intestinal mucosal injury in IBD with epithelial attachment loss in periodontitis.^1^, ^17^

Furthermore, in vitro co‐culture systems have revealed critical interactions between neutrophils and other cell types, including APCs, fibroblasts, and epithelial cells. NET components have been shown to activate mitogen‐activated protein kinase (MAPK) pathways, particularly ERK1/2, within IECs, leading to increased transcription of pro‐inflammatory cytokines such as TNF‐α and IL‐1β. These effects are reversible with pharmacological inhibition of NET formation, using DNase or PAD4 inhibitors, highlighting a direct mechanistic link between NETs and epithelial inflammatory responses.^156^, ^157^, ^158^, ^159^, ^160^, ^161^, ^162^

In addition, fibroblasts isolated from therapy‐resistant IBD lesions have been shown to produce elevated levels of chemokines CXCL1, CXCL2, and CXCL8 (IL‐8) in response to IL‐1 receptor signaling. This creates a feed‐forward loop wherein neutrophil chemotaxis is promoted, further amplifying local inflammation.^163^ Another important mechanism involves immunoglobulin G (IgG) immune complexes binding to neutrophil Fc gamma receptors (FcγRs), including CD64, CD32, and CD16. This binding is significantly upregulated in active IBD and enhances neutrophil respiratory burst activity and the release of additional pro‐inflammatory mediators.^164^ Collectively, in vitro data underscore how neutrophils contribute to mucosal barrier dysfunction, promote epithelial injury via NET release, and engage in complex cellular crosstalk that perpetuates intestinal inflammation.

#### 3.4.3 Animal models

Animal models have been instrumental in establishing the biological plausibility of neutrophil involvement in IBD. The dextran sulfate sodium (DSS)‐induced colitis model in mice is one of the most widely used models of UC‐like disease, characterized by epithelial injury and robust neutrophilic infiltration.^165^ Recently, Wang et al.^166^ investigated the role of PAD4 in IBD, focusing on its enzymatic activity in citrullination and influence on intestinal inflammation. Using single‐cell RNA sequencing (scRNA‐seq) and citrullination mapping in a DSS‐induced colitis mouse model, the authors found that PAD4 deficiency reduced colonic inflammation, restored the intestinal barrier, and altered both immune and epithelial cell populations, particularly by decreasing neutrophil infiltration and modifying epithelial gene expression profiles associated with inflammation. A key mechanistic finding was that neutrophil‐derived EVs delivered PAD4 into IECs. Once inside IECs, PAD4 citrullinated mitochondrial creatine kinase 1 (CKMT1) at residue R242, reducing CKMT1 protein stability through autophagy. This disrupted mitochondrial function, compromised epithelial integrity, and triggered apoptosis. Depletion of CKMT1 in IECs further worsened inflammation and epithelial damage in colitis models. Importantly, clinical samples from IBD patients showed increased PAD4 expression, enhanced CKMT1 citrullination, and decreased CKMT1 protein levels, linking the findings to human disease.^166^

In acute DSS‐induced colitis models in both mice and rats, pharmacological or antibody‐mediated depletion of neutrophils, using agents such as anti‐Ly6G or anti‐Gr‐1 antibodies, consistently resulted in a marked attenuation of disease severity. Treated animals exhibited less weight loss, reduced diarrhea and rectal bleeding, diminished histologic evidence of epithelial damage, and preserved colonic architecture and length.^156^, ^157^, ^159^, ^162^, ^164^ These findings strongly implicate neutrophils in the propagation of acute colonic inflammation. However, it is important to note that in certain experimental contexts, neutrophil depletion has paradoxically led to disease exacerbation, reflecting the complex and context‐dependent roles neutrophils may play in tissue homeostasis and repair.

NET formation has emerged as a hallmark of experimental colitis. DSS‐treated animals display upregulation of PAD4 expression, histone H3 citrullination, increased MPO activity, and elevated levels of circulating cell‐free DNA, features that collectively indicate robust NETosis.^156^ Therapeutic strategies targeting NET formation, such as systemic DNase administration, PAD4 inhibition, or blockade of neutrophil elastase, have been shown to ameliorate colonic mucosal injury, restore epithelial barrier function, and suppress expression of pro‐inflammatory cytokines. In colitis models, similar observations have been made, with NET‐associated neutrophil accumulation correlating with epithelial apoptosis and mucosal ulceration. Inhibiting NETosis in these settings consistently reduced disease severity and histological damage.^156^

The dynamic interplay between neutrophils, cytokines, and other innate immune cells has also been elucidated in murine models. For example, blockade of GM‐CSF in colitis led to reduced neutrophil infiltration, lower ROS generation, decreased IL‐1β production, and downregulation of activation markers such as CD11b and CD62L on circulating neutrophils, culminating in improved clinical and histological outcomes.^167^ Additionally, IL‐23 has been shown to skew neutrophils toward the secretion of IL‐17 and IL‐22, endowing them with pathogenic T‐cell‐like functions. Adoptive transfer of IL‐23‐conditioned neutrophils exacerbated colitis in recipient mice in an IL‐17–dependent manner; notably, this effect was attenuated in IL‐17A‐deficient animals.^157^, ^158^, ^159^, ^160^, ^162^, ^167^

Moreover, regulatory crosstalk between neutrophils and NK cells has been demonstrated. In colitis, NK cells exert anti‐inflammatory effects through NKG2A‐mediated interactions that suppress neutrophil ROS production and cytokine release, while simultaneously promoting IL‐10 and IL‐4 expression. NK cell depletion abrogates this regulation, resulting in heightened neutrophil‐driven inflammation and worsened colitis.^159^

Importantly, the effects of colitis extend beyond the intestinal mucosa. It induces systemic neutrophilia and promotes neutrophil infiltration into distal organs such as the lungs, where they contribute to secondary inflammation and increased susceptibility to bacteremia. This is largely mediated by IL‐6–dependent mobilization of neutrophils from the bone marrow.^161^ In autoimmune‐prone nonobese diabetic (NOD) mice, DSS exposure—which experimentally induces colitis—augments systemic NET formation, not only within the colon but also in peripheral tissues. This heightened neutrophil activation promotes the expansion of Th1 and Th17 cells, accelerating the onset of type 1 diabetes and underscoring the systemic immunological consequences of colitis.^158^

Finally, studies have shown that neutrophils can compensate for deficiencies in other innate phagocytes. In experimental models where macrophages or DCs are selectively depleted, neutrophil recruitment increases as a compensatory mechanism.^168^, ^169^ However, this compensatory influx often exacerbates inflammation and tissue damage. Notably, combined depletion of neutrophils with other innate immune cells mitigates disease severity, suggesting that neutrophils can amplify inflammation in the absence of regulatory cues from other cell types.

Taken together, the integration of findings from in vitro systems and animal models provides compelling biological plausibility for the central role of neutrophils in IBD pathogenesis. In vitro experiments elucidate how neutrophil‐derived enzymes, NET components, and Fcγ receptor engagement directly compromise epithelial integrity, disrupt mucosal homeostasis, and propagate pro‐inflammatory signaling. Animal models corroborate these mechanistic insights by demonstrating that genetic or pharmacologic targeting of neutrophil activity, especially NETosis and cytokine polarization, ameliorates disease severity, preserves barrier function, and restores immune equilibrium. Furthermore, the plasticity of neutrophils under the influence of cytokine and stromal cues highlights their multifaceted role in either promoting or resolving inflammation.

These findings collectively position neutrophils not only as critical effectors in mucosal immunity but also as potential therapeutic targets. Future studies should focus on the development of strategies that selectively modulate neutrophil trafficking, NET release, and effector functions, while preserving their essential roles in antimicrobial defense. However, it is also important to acknowledge that such animal studies may not directly translate to human IBD and periodontal comorbidities. The main reasons are that DSS‐induced colitis models differ from IBDs, which have multifaceted risk factors, causative agents, and variable disease progression. Moreover, the human gut microbiome differs from that of rodents,^170^ and immune responses are mediated by different immune cell ratios compared with humans, particularly with regard to neutrophils.^171^

## 4 RETRIEVAL AND PHENOTYPING OF NEUTROPHILS IN IBD IN HUMANS

In humans, evidence on neutrophil phenotyping and characterization during IBD derives primarily from PBN isolation. Although PBNs are relatively simple to isolate and study functionally, their stage of differentiation and behavior, being derived from a mostly sterile compartment such as the bloodstream, may differ from that observed in the GI mucosa. In this section, we describe potential sources for neutrophil retrieval and report findings in CD and UC in humans.

### 4.1 Peripheral blood

Peripheral blood is probably the most well reported source of neutrophils for studies in IBD. Microscopic examination of toluidine blue‐stained preparations of peripheral venous blood showed that active CD and UC are related to a reduced proportion of spherical inactivated circulating neutrophils compared with their inactive counterpart and healthy controls.^172^ Patients with UC expressed higher neutrophil counts than controls, and FC levels demonstrated a correlation with UC's clinical activity index and mucosal inflammation.^173^ Periodontal research has similarly relied upon PBNs to demonstrate systemic priming effects of chronic oral inflammation, supporting the use of circulating neutrophil phenotypes as a shared readout across intestinal and oral inflammatory diseases.^1^, ^15^, ^22^

Patients with active IBD expressed more CD177+ neutrophils in peripheral blood compared with healthy controls and were associated with gene expression profiles related to bacterial defense responses, hydrogen peroxide, and ROS.^174^ This subset of neutrophils expressed lower levels of pro‐inflammatory cytokines. In contrast, IL‐22, TGF‐β, ROS, antimicrobial peptides, and NETs were more expressed in CD177+ than CD177− subsets.^174^ TNF‐α treatment appears to promote NET release in UC patients' circulating neutrophils.^155^ In pediatric IBD patients, PBNs from UC patients exhibited increased NET formation compared with controls and CD patients, which normalized following remission‐inducing treatment.^175^

IFN‐α seems to play an important role in neutrophil responses in CD, as opposed to UC. High IFΝ‐responsive signatures in peripheral neutrophils were observed in CD, distinguishing it from UC.^176^ Moreover, CD serum stimulated the release of fibrogenic‐enriched NETs from neutrophils in an IFN‐α‐dependent manner, suggesting a priming role of IFN‐α in circulating neutrophils, something that has been previously demonstrated in PBNs from periodontitis patients.^177^ Furthermore, both serum IFN‐α levels and mRNA levels of key IFN signaling components in neutrophils were well correlated with CD severity.^176^ Notably, chronic periodontal inflammation has also been associated with systemic priming of circulating neutrophils, including heightened responsiveness to inflammatory cytokines and type‐1 IFN‐related signaling pathways, suggesting that the oral inflammatory burden may modulate peripheral neutrophil phenotypes relevant to the IFN‐driven mechanisms observed in IBD.^15^, ^20^, ^22^

PBNs from CD patients in remission showed normal IL‐8‐induced migration, phagocytosis and signaling, but impaired transepithelial migration and increased N‐formyl‐methionyl‐leucyl‐phenylalanine (fMLP)‐stimulated ROS production compared with healthy controls.^178^ Rates of end‐stage apoptotic neutrophils were not significantly different between CD and controls, despite decreased cleavage of caspase‐3 and caspase‐8 during MCSF‐induced rescue from cell death in CD neutrophils.^178^

### 4.2 Saliva

IBD is not restricted to the intestines, and the oral cavity may also be affected.^179^ Therefore, oral neutrophils collected from saliva samples could be a potential source for neutrophil biology studies in IBD. However, data on neutrophil harvesting in IBD patients is limited. One study used PBS‐diluted stimulated saliva (chewing on 0.5 g of paraffin for 5–10 min) filtered through 70, 40, and 20 μm filters to analyze phenotype by fluorescence‐activated cell sorting (FACS) and calprotectin expression by immunofluorescence (intracellular amount) and ELISA (supernatant) after 1 or 3 h‐culture.^180^ The frequency of CD15+CD16+ cells and CD15 or CD16 expression was not statistically different in IBD patients and controls. CD11b, on the other hand, was significantly reduced on neutrophils from IBD patients compared with controls, particularly in CD compared with UC. Immunofluorescence revealed cytosolic calprotectin in salivary neutrophils, and cells from the control group tended to secrete less calprotectin after both 1 and 3 h. IBD patients tended to have significantly less calprotectin in cell lysates than in the corresponding supernatant, but the same pattern was not observed for controls.^180^ From a periodontal research perspective, saliva and oral neutrophils are extensively employed as a noninvasive sampling source to investigate tissue‐experienced neutrophil phenotypes and local inflammatory activity within the oral cavity.^1^, ^19^, ^180^

### 4.3 Biopsy

Biopsies can be a practical tool for characterizing neutrophil infiltrates in IBD patients undergoing colonoscopy. Neutrophils and NETs are highly expressed in biopsies from UC and CD patients when compared with controls, and this association is stronger in UC patients.^175^ One study analyzing the predictive role of Claudin‐2 and junctional adhesion molecule A (JAM‐A) in IBD patients showed that neutrophils in the epithelium significantly predicted adverse outcomes for both UC and CD (hazard ratio 5.2 and 4.4, respectively). Claudin‐2 correlated with histological activity and predicted outcomes in UC, while JAM‐A showed lower specificity in those patients.^181^

The response of neutrophils in CD in children seems to be directed, at least in part, against luminal enterobacteria and enterocytes.^182^ This was shown when evaluating surgical resections and endoscopic biopsies from the ileum using light and electron microscopy. Additionally, different treatments may modulate neutrophil infiltrates in gut mucosa. In CD, a 4‐week treatment with infliximab increased monocytes and lymphocytes moderately, while neutrophils were decreased.^183^ Additionally, NET‐associated proteins were over‐expressed in inflamed colons of UC patients as compared with CD patients and NC.^155^

Comparable biopsy‐based approaches are routinely employed in periodontal research, where gingival tissue samples enable in situ assessment of neutrophil infiltration, NET formation, and tissue‐specific inflammatory signatures. Gingival biopsies have been widely used to characterize the spatial distribution of neutrophils within the junctional epithelium and connective tissue, to evaluate their interactions with epithelial, stromal and collaborating immune cells, and to quantify the expression of neutrophil‐derived mediators, such as MPO, neutrophil elastase, and citrullinated histones. In addition, periodontal tissue biopsies allow detailed analysis of local immune–microbial interactions, including the relationship between neutrophil activation states, microbial dysbiosis, and tissue destruction at disease sites.^1^, ^18^, ^21^ Together, these biopsy‐based strategies provide complementary, site‐specific insights into tissue‐experienced neutrophil phenotypes, closely paralleling the use of intestinal biopsies to study mucosal neutrophil behavior in IBD.

### 4.4 Stool

Analyses of stool samples are frequently related to products of leukocytes, including neutrophils, but data is limited when it comes to neutrophil retrieval, counting, and characterization. In pediatric patients, the neutrophil/lymphocyte ratio was significantly correlated to FC, clinical activity score, and Mayo endoscopic subscore.^184^ In adults, FC levels correlated with the endoscopic and clinical disease activities and laboratory parameters, including serum levels of hemoglobin (Hb), albumin, and CRP, and ESR, particularly among the patients with UC. Both UC and CD had higher neutrophil and monocyte/macrophage calprotectin‐positive cell expression levels compared with healthy controls.^185^ Stool analysis also showed the predominant site of neutrophil chemoattractants to be within the mucosa in UC, while within the lumen in CD.^186^ As a functional analog of stool‐based inflammatory readouts, gingival crevicular fluid provides a site‐specific, neutrophil‐rich fluid for assessing inflammatory mediators, such as calprotectin, MPO, and elastase.^1^, ^18^, ^21^

### 4.5 Whole gut lavage

Whole gut lavage fluid has been proposed as a method to analyze luminal neutrophils and their products. For that purpose, patients take a bowel cleansing fluid such as polyethylene glycol‐based products, which is widely used prior to colonoscopy.^187^ Then, when the rectal effluent is clear, it can be analyzed for total cell count, neutrophil count, and neutrophil‐derived products. Cells are separated by density gradient centrifugation, counted, and cytospin preparations can be examined.^35^ Granulocyte elastase was significantly correlated with cell count, although not all patients presented with high levels of this enzyme in the fluid.^35^ In fact, not only granulocyte elastase, but also IL‐8 and IL‐1β levels can differ according to colonic and ileal disease distribution.^33^

In periodontal research, comparable lavage‐based approaches are used to sample the oral mucosal interface, most commonly through gingival sulcus lavage. This technique typically involves gentle irrigation of the gingival crevice with a defined volume of sterile isotonic solution, followed by immediate aspiration or collection of the lavage fluid, allowing recovery of neutrophils, soluble inflammatory mediators, and microbial products from the periodontal niche.^18^, ^21^ Gingival sulcus lavage has been applied to quantify neutrophil‐derived enzymes, cytokines, and lipid mediators, as well as to assess local inflammatory burden and disease activity in a site‐specific and minimally invasive manner.^1^, ^18^ As with gut lavage in IBD, oral lavage‐based sampling provides access to luminal or interface‐associated immune components that are not readily captured through tissue biopsies or peripheral blood analysis.^1^, ^21^

### 4.6 Colonoscopic aspirate

Although colonoscopic aspirate is easy to collect and allows for the analysis of neutrophils in situ, to the best of our knowledge, to date it has only been used to isolate *Escherichia coli*.^188^ Our group is currently working on the standardization of aspirate collection protocols that enable the study of neutrophils that have transmigrated into the gut lumen.^189^ Our preliminary results suggest that we can recover viable cells exhibiting markers related to advanced stages of differentiation and compromised ROS production.

The periodontal research counterpart of this technique involves aspirate‐based and micro‐sampling approaches, including gingival sulcus aspirates and crevicular fluid collection using paper strips or, historically, microcapillary tubes, which are well established and provide complementary strategies to study locally activated neutrophils.^1^, ^18^

## 5 DEVELOPMENT OF NEW PROTOCOLS FOR STUDYING NEUTROPHILS IN IBD AND/OR PERIODONTITIS IN HUMANS

Taken together, the parallel use of blood‐, luminal‐, and tissue‐based sampling approaches in intestinal and periodontal research provides a framework for integrated oral‐gut neutrophil profiling in future translational studies. Coordinated analysis of circulating neutrophils alongside site‐specific oral (e.g., gingival crevicular fluid, oral lavage) and intestinal samples could enable direct comparison of neutrophil activation profiles and functional states across the oral and gut mucosa. Such approaches would allow evaluation of tissue‐specific neutrophil responses to inflammatory cues in the oral cavity and the intestine, facilitating comparison of activation levels, trafficking behavior, and resolution capacity in distinct mucosal environments. Ultimately, integrated sampling strategies may help clarify how chronic oral inflammation contributes to systemic neutrophil priming and modulates neutrophil phenotypes relevant to intestinal disease.

**FIGURE 4:**
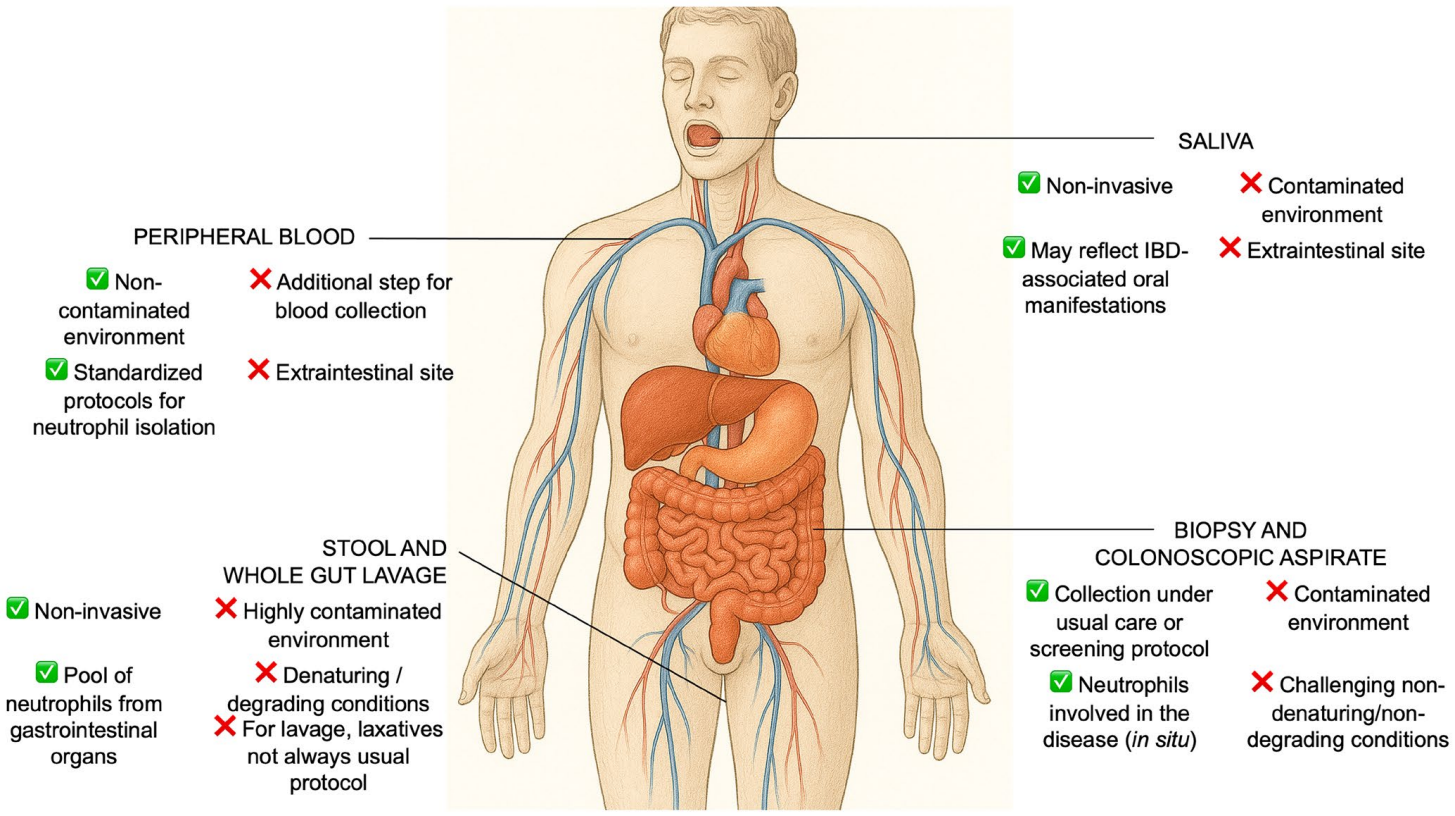
Summary of the advantages (checks in green) and limitations (crosses in red) of each current harvesting site for studying neutrophils in inflammatory bowel disease (IBD) in humans.

An ideal protocol for analyzing the characteristics of cells in IBD includes harvesting in proximity to the inflamed site; a low contamination environment, when possible; use of nondegrading and nondenaturing buffers or conditions; rapid sample processing; noninvasive harvesting or collection under usual care; and minimal side effects. Therefore, as can be inferred from the potential biological sources of neutrophils listed in the section “Retrieval and Phenotyping of Neutrophils in IBD in Humans,” biopsies and aspirates offer the most potential for an ideal protocol, although local contamination remains an issue.^189^ A summary of the advantages and limitations of each current harvesting site is shown in Figure 4.

A range of analytical modalities is now available to interrogate neutrophil biology across the distinct compartments relevant to IBD and periodontal disease, each with specific advantages and technical limitations. In peripheral blood, intestinal lavage, or gingival samples, flow cytometry immunophenotyping remains the most feasible approach for quantifying neutrophil subsets, activation markers, and FcγR expression, although rapid processing is essential to avoid ex vivo activation and loss of surface epitopes.^136^ scRNA‐seq of intestinal or periodontal biopsies enables high‐resolution profiling of neutrophil transcriptional states within the tissue microenvironment, but a number of technical challenges remain due to the fragile nature of neutrophils, including low RNA content and susceptibility to activation during enzymatic dissociation.^140^ Functional assays, including ROS production, NET formation, chemotaxis, and phagocytosis, can be performed on neutrophils isolated from blood, lavage, or tissue aspirates, yet, these assays are highly sensitive to cell viability, microbial contamination, and the presence of epithelial debris, factors particularly relevant in mucosal tissues with high microbial load.^135^ In both intestinal and periodontal compartments, sample processing steps such as mechanical disruption, digestion, and prolonged handling can induce transcriptional and functional artifacts, underscoring the need for standardized protocols that minimize activation and preserve physiological neutrophil states.^143^ Collectively, these methodological considerations highlight the importance of selecting compartment‐appropriate analytical platforms while acknowledging the inherent technical pitfalls that may influence interpretation of neutrophil phenotypes in chronic mucosal inflammation.

The rationale for developing new protocols for neutrophil phenotyping in IBD patients includes building databases that may cluster patients according to their diagnosis and prognosis, and help to define monitoring protocols and therapeutic care pathways more consistent with a personalized and precision approach to care.

## 6 CONCLUDING REMARKS

Neutrophils represent a dynamic and context‐dependent component of the inflammatory landscape in IBD. Their dual capacity to exacerbate mucosal damage or promote resolution underscores the need for improved methodological approaches that enable precise characterization of their functional states in both systemic and tissue contexts. A refined understanding of neutrophil heterogeneity and behavior may reveal novel insights into disease mechanisms and identify new therapeutic targets.

Clarifying the diverse roles and activation states of neutrophils in IBD has significant clinical implications: (1) Improved phenotypic and functional profiling could facilitate the identification of biomarkers predictive of disease activity, treatment response, and relapse risk; (2) Targeting specific neutrophil subsets or effector pathways may open new avenues for precision medicine approaches aimed at restoring immune balance and promoting mucosal healing in patients with CD and UC; (3) Shed light on their specific roles in the interplay between IBD and other diseases, including periodontitis; and (4) Map subsets and their respective functions in neutrophils' natural history, from peripheral blood to terminal tissues such as the intestines, saliva, or gingival crevicular fluid, possibly correlating with disease severity.

## FUNDING INFORMATION

RSM is currently supported by grant provided by Sao Paulo Research Foundation (FAPESP, São Paulo, Brazil), award #2023/15750‐7. Part of the research reported here was funded by the National Institute for Health and Care Research (NIHR) Birmingham Biomedical Research Centre (BRC) (Grant #NIHR2023326). The views expressed are those of the author(s) and not necessarily those of the NIHR or the Department of Health and Social Care.

## CONFLICT OF INTEREST STATEMENT

All authors declare they have no conflict of interest related to this study.

## Data Availability

Data sharing not applicable to this article as no datasets were generated or analyzed during the current study.
